# Respiratory syncytial virus bronchiolitis complicated by spontaneous pneumothorax in an infant: a case report

**DOI:** 10.11604/pamj.2025.52.67.49324

**Published:** 2025-10-09

**Authors:** Hajar Eddukar, Sara Aminou, Soumia Benchekroun, Chafiq Mahraoui, Naima ElHafidi

**Affiliations:** 1 Department of Pediatric Pneumo-Allergology and Infectious Diseases, Children’s Hospital, Ibn Sina University Hospital, Faculty of Medicine and Pharmacy, Mohammed V University, Rabat, Morocco

**Keywords:** Bronchiolitis, respiratory syncytial virus, infant, pneumothorax, case report

## Abstract

Acute bronchiolitis is the most common viral lower respiratory tract infection in infants, with an annual incidence of 3-5% in children under 12 months, peaking between 2 and 6 months of age. Respiratory syncytial virus (RSV) is responsible for 50-80% of cases. Although generally benign, bronchiolitis may be complicated by spontaneous pneumothorax, which is reported in only 0.5-2% of hospitalized infants, making it a rare but potentially life-threatening event. We report the case of an 11-month-old female infant with a family history of maternal asthma, admitted for acute respiratory distress during a second episode of bronchiolitis. Examination revealed tachypnea, wheezing, and retractions. RSV infection was confirmed by polymerase chain reaction. Imaging showed a moderate left-sided pneumothorax with partial atelectasis. Conservative management with oxygen, nebulized salbutamol, and intravenous corticosteroids led to full recovery without invasive intervention. This rare case emphasizes the importance of considering pneumothorax in bronchiolitis with sudden deterioration. In stable infants, conservative treatment may be sufficient.

## Introduction

Acute bronchiolitis is the leading cause of lower respiratory tract infection and hospitalization in infants, with an annual incidence of 3-5% in children under one year, peaking between 2 and 6 months of age [1,2]. Respiratory syncytial virus (RSV) is implicated in 50-80% of cases, making it the predominant etiological agent [1,3]. While bronchiolitis is usually self-limiting, it may lead to serious complications such as respiratory failure, apnea, bacterial superinfection, and, more rarely, air leak syndromes including pneumothorax or pneumomediastinum [4,5]. Spontaneous pneumothorax in the context of bronchiolitis is exceptionally uncommon, with a reported incidence of 0.5-2% among hospitalized infants [5,6]. Its occurrence may dramatically worsen the clinical course and, although bronchiolitis carries an overall low mortality (<1% in developed countries), outcomes are more severe in high-risk infants, such as those born prematurely or with congenital heart or lung disease [2,7].

Given its rarity, only a few cases of bronchiolitis complicated by pneumothorax have been described in the literature. We report here the case of an infant with RSV bronchiolitis complicated by spontaneous pneumothorax, which resolved favorably with conservative management, underlining both the potential severity of this complication and the possibility of a non-invasive therapeutic approach.

## Patient and observation

**Patient information:** we report the case of an 11-month-old female infant, the second child of non-consanguineous parents, born at term after an uneventful pregnancy and delivery, with normal growth and psychomotor development. She had been on mixed feeding since birth, with dietary diversification introduced at 6 months, and was well-tolerated. Her history included hospitalization at 9 months for acute bronchiolitis, followed by an asymptomatic intercritical interval. One month prior to admission, she presented a choking episode with transient cyanosis that resolved spontaneously. Family history was significant for maternal asthma, but no congenital or chronic lung disease. Immunizations were up-to-date, the infant was not exposed to tobacco smoke, and she lived in a stable socio-economic setting.

**Clinical findings:** on admission, the patient was in moderate respiratory distress. She was afebrile, hemodynamically stable, and presented with tachypnea, intercostal and subcostal retractions, and expiratory wheezing. Peripheral oxygen saturation was 91% in room air. Cardiac and systemic examinations were otherwise normal.

**Figure 1:**
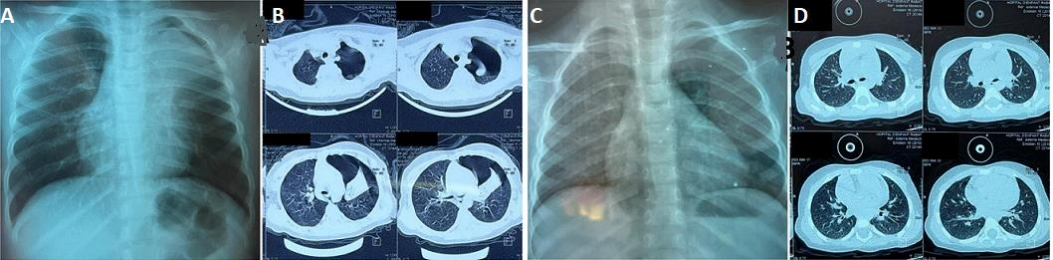
(A,B) left pneumothorax, apical atelectasis of the left lung, chest radiography and chest computed tomography; (C,D) resolution of pneumothorax with good pulmonary expansion, chest radiography and chest computed tomography

**Timeline of current episode:** at presentation (00h), the infant had a respiratory rate of 57 breaths/min with signs of moderate respiratory distress, including intercostal retractions and nasal flaring. Auscultation revealed diffuse expiratory wheezing, more pronounced at the lung bases. Percussion was normal, without hyperresonance or dullness. Oxygen saturation was 91% on room air. Chest expansion was symmetrical, and vocal fremitus was normal. At 6h, under oxygen supplementation and bronchodilators, oxygen saturation increased to 93%, with persistent retractions and wheezing. At 12h, clinical worsening occurred with tachypnea (68 breaths/min), desaturation (88% despite oxygen therapy), and nasal flaring. Breath sounds decreased at the left apex, and chest radiography confirmed a moderate left pneumothorax. At 24h, with conservative management and continuous monitoring, oxygen saturation normalized (96% in room air) and respiratory rate decreased to 45/min. At 48h, recovery was almost complete, with resolution of distress and normal auscultation. Chest radiography confirmed re-expansion of the left lung (Figure 1). At discharge (day 6), the infant was clinically stable, with normal examination and oxygen saturation of 99% in room air.

**Diagnostic assessment:** nasopharyngeal swab confirmed respiratory syncytial virus infection by molecular testing using polymerase chain reaction. Laboratory tests showed mild inflammation (CRP 17 mg/L) and normal blood counts (Hb 11.8 g/dL, WBC 10,200/mm^3^, platelets 320,000/mm^3^). No bacterial coinfection was identified. Initial chest X-ray revealed diffuse bronchial syndrome without consolidation. At 12h, repeat imaging chest X-ray showed an avascular apico-lateral hyperlucency of the left lung, with a left pericardial opacity corresponding to the retracted lung, consistent with a moderate left-side pneumothorax. Thoracic computed tomography without contrast administration showed a moderate left-sided pneumothorax associated with partial left apical atelectasis, ruling out pneumonia, congenital malformation, or foreign body aspiration (Figure 1).

**Diagnosis:** the final diagnosis was RSV bronchiolitis complicated by a spontaneous left-sided pneumothorax with apical atelectasis.

**Therapeutic interventions:** management included oxygen therapy (0.5 L/min), nebulized salbutamol, and intravenous methylprednisolone, in line with international guidelines for recurrent bronchiolitis with atopic predisposition. Following pneumothorax onset, the patient was closely monitored. Conservative treatment was maintained without the need for decompression or chest drainage, as the pneumothorax remained moderate, and the clinical course improved progressively.

**Follow-up and outcomes:** the patient improved steadily over 48h, with normalization of respiratory function and radiographic resolution of the pneumothorax. She was discharged after six days in good general condition. At 6-month follow-up, she remained asymptomatic, with appropriate growth and no pulmonary sequelae.

**Patient perspective:** the parents initially expressed anxiety about the pneumothorax and the potential need for invasive procedures, but were reassured by the favorable outcome under conservative management. They emphasized their awareness of preventive care and follow-up in view of the family´s atopic history.

**Informed consent:** written informed consent was obtained from the parents for publication of this case and the associated clinical and imaging data.

## Discussion

Acute bronchiolitis is the most common viral lower respiratory tract infection in infants and represents the leading cause of hospitalization during the first year of life worldwide. Its annual incidence is estimated between 3 and 5% among infants under 12 months of age, with the highest burden between two and six months [1,2]. Respiratory syncytial virus (RSV) is responsible for the majority of cases, accounting for 50 to 80% of episodes [1,3]. In our patient, bronchiolitis was due to RSV, confirmed by polymerase chain reaction, and occurred during the first year of life at 11 months, corresponding to the typical epidemiological profile. Although mortality in bronchiolitis is usually low (<1% in high-income countries), it can increase in severe cases and when complications occur [2,7]. Our case illustrates this potential severity, since the child presented a rare but potentially life-threatening complication. The pathophysiology of bronchiolitis involves viral invasion of the bronchiolar epithelium, with mucosal edema, mucus hypersecretion, and airway obstruction. Respiratory syncytial virus infection generates intense neutrophilic infiltration, release of IL-6, IL-8, TNF-α, and proteolytic enzymes, contributing to epithelial fragility [8]. These mechanisms explain the obstructive syndrome observed in our patient, who presented with tachypnea, wheezing, and retractions. The high respiratory rate (68 breaths/min) and expiratory braking likely promoted alveolar hyperinflation, eventually leading to rupture and pneumothorax [5,6].

Spontaneous pneumothorax complicating bronchiolitis is rare, with a prevalence of 0.5-2% among hospitalized infants [5,6]. It usually occurs in younger infants or those with severe obstruction or atopic background [7]. Our patient was 11 months old, female, with a family history of atopy (maternal asthma), which corresponds to several risk factors described in the literature. Clinically, she developed acute respiratory distress with hypoxemia and decreased breath sounds on the left, suggesting an air-leak syndrome. This highlights the importance of considering pneumothorax in any bronchiolitis patient with sudden clinical deterioration. Imaging remains the cornerstone of diagnosis. Chest X-ray is the first-line investigation and confirmed a moderate left-sided pneumothorax in our patient. Computerized tomography (CT) was performed due to suspicion of pneumonia or foreign body aspiration, confirming the diagnosis and excluding other causes. This use of CT is consistent with recommendations to limit its indications to atypical or doubtful cases [6]. The finding of a localized left pneumothorax with partial atelectasis matched the clinical presentation of unilateral decreased breath sounds. Management depends on pneumothorax size and clinical tolerance. In severe or large cases, needle decompression or drainage may be lifesaving. However, these interventions carry risks such as infection, persistent air leak, or iatrogenic lung injury [3,9]. In our case, despite the presence of a moderate pneumothorax, the patient remained stable, allowing a conservative strategy with oxygen, salbutamol nebulizations, and intravenous corticosteroids. This choice avoided unnecessary invasive procedures.

The monitoring protocol, initially hourly, then every four hours after stabilization, ensured safe observation. The evolution in our case supports data from the literature showing that spontaneous resolution can occur in stable infants [6]. The control chest X-ray after 48 hours showed complete re-expansion, and the patient was discharged after six days without sequelae. At six months of follow-up, she remained asymptomatic. This favorable outcome emphasizes that, under strict monitoring, conservative management can be successful. Comparison with previously reported (Table 1) cases reveals common points. The majority of published infants with bronchiolitis-related pneumothorax were between 3 and 14 months of age, and many were females, like our patient [3,9]. Respiratory syncytial virus was the most frequent agent, and treatment strategies varied, ranging from observation to drainage. Some infants required invasive procedures, while others, as in our case, improved without intervention. This highlights the need for individualized evaluation, taking into account both clinical stability and radiological findings. Our observation demonstrates that pneumothorax complicating bronchiolitis, although rare, should always be suspected when clinical worsening occurs. Early radiographic confirmation, close monitoring, and individualized management allowed spontaneous resolution without invasive intervention. The absence of recurrence after six months of follow-up supports the long-term safety of this approach. This case thus reinforces the importance of vigilance in bronchiolitis and contributes to the literature by illustrating a favorable outcome under conservative treatment.

**Table 1:** presents a summary of reported cases of spontaneous pneumothorax associated with bronchiolitis

Authors	Number of cases	Age (months)	Sex	Effusion type	Onset delay (days)	Agent	Treatment
Lipinski and Goodman	1	8	Female	PNX	3	RSV	Chest drainage
Pollack	1	4	Female	PNX -pneumomediastinum	1	RSV	Needle decompression
Alter	1	4	Male	PNX	2	ND	Assisted ventilation and chest drainage
Piastra *et al*.	2	6/11	Female	PNX-SCE	2 / 4-5	RSV	Observation
Kambouri *et al*.	1	3	Female	PNX	7	RSV	Needle decompression
Given *et al*.	4	5 / 10 / 5 / 24	F/M/F/M	PNX/Pneumomediastinum-SCE	3/4/3/3	RSV/Parainfluenza/ND	Observation
Hopkins *et al*.	1	14	Male	SCE	2	RSV	Observation
Tutor *et al*.	1	9	Female	Pneumomediastinum-SCE	10	Influenza A	Observation
**Our case**	1	11	Female	PNX	3	RSV	Observation

Legend: PNX: pneumothorax; ESC: subcutaneous emphysema; ND: not determined; RSV: respiratory syncytial virus.

## Conclusion

Pneumothorax in bronchiolitis, though uncommon, is potentially life-threatening. Prompt recognition and careful conservative management can lead to full recovery without invasive procedures, underscoring the need for clinical awareness of this rare complication.

## References

[1] Hall CB (2001). Respiratory syncytial virus and parainfluenza virus. N Engl J Med.

[2] Ralston SL, Lieberthal AS, Meissner HC, Alverson BK, Baley JE, Gadomski AM (2014). Clinical practice guideline: the diagnosis, management, and prevention of bronchiolitis. Pediatrics.

[3] Lipinski JK, Goodman D (1980). Pneumothorax complicating bronchiolitis in infancy: a case report. Pediatr Radiol.

[4] Pollack MM, Fields AI, Holbrook PR (1979). Pneumothorax and pneumomediastinum during pediatric mechanical ventilation. Crit Care Med.

[5] Alter SJ (1997). Spontaneous pneumothorax in infants: a 10-year review. Pediatr Emerg Care.

[6] Piastra M, Caresta E, Tempera A, Langer A, Zorzi G, Pulitanò S (2006). Sharing features of uncommon respiratory syncytial virus complications in infants. Pediatr Emerg Care.

[7] Kambouri K, Gardikis S, Tsalkidis A, Cassimos D, Deftereos S, Chatzimichael A (2007). Late onset of spontaneous pneumothorax complicating acute bronchiolitis in a 5-month-old infant: case report and literature review. Pediatr Emerg Care.

[8] Openshaw PJM, Chiu C (2013). Protective and dysregulated T cell immunity in RSV infection. Curr Opin Virol.

[9] Tutor JD, Montgomery VL, Eid NS (1995). A case of influenza virus bronchiolitis complicated by pneumomediastinum and subcutaneous emphysema. Pediatr Pulmonol.

